# MRI active guidewire with an embedded temperature probe and providing a distinct tip signal to enhance clinical safety

**DOI:** 10.1186/1532-429X-14-38

**Published:** 2012-06-21

**Authors:** Merdim Sonmez, Christina E Saikus, Jamie A Bell, Dominique N Franson, Majdi Halabi, Anthony Z Faranesh, Cengizhan Ozturk, Robert J Lederman, Ozgur Kocaturk

**Affiliations:** 1 Division of Intramural Research, National Heart Lung and Blood Institute, National Institutes of Health, Bethesda, MD 20892, USA; 2 Institute of Biomedical Engineering, Bogazici University, Istanbul, Turkey

## Abstract

**Background:**

The field of interventional cardiovascular MRI is hampered by the unavailability of active guidewires that are both safe and conspicuous. Heating of conductive guidewires is difficult to predict in vivo and disruptive to measure using external probes. We describe a clinical-grade 0.035” (0.89 mm) guidewire for MRI right and left heart catheterization at 1.5 T that has an internal probe to monitor temperature in real-time, and that has both tip and shaft visibility as well as suitable flexibility.

**Methods:**

The design has an internal fiberoptic temperature probe, as well as a distal solenoid to enhance tip visibility on a loopless antenna. We tested different tip-solenoid configurations to balance heating and signal profiles. We tested mechanical performance in vitro and in vivo in comparison with a popular clinical nitinol guidewire.

**Results:**

The solenoid displaced the point of maximal heating (“hot spot”) from the tip to a more proximal location where it can be measured without impairing guidewire flexion. Probe pullback allowed creation of lengthwise guidewire temperature maps that allowed rapid evaluation of design prototypes. Distal-only solenoid attachment offered the best compromise between tip visibility and heating among design candidates. When fixed at the hot spot, the internal probe consistently reflected the maximum temperature compared external probes.

Real-time temperature monitoring was performed during porcine left heart catheterization. Heating was negligible using normal operating parameters (flip angle, 45°; SAR, 1.01 W/kg); the temperature increased by 4.2°C only during high RF power mode (flip angle, 90°; SAR, 3.96 W/kg) and only when the guidewire was isolated from blood cooling effects by an introducer sheath. The tip flexibility and in vivo performance of the final guidewire design were similar to a popular commercial guidewire.

**Conclusions:**

We integrated a fiberoptic temperature probe inside a 0.035” MRI guidewire. Real-time monitoring helps detect deleterious heating during use, without impairing mechanical guidewire operation, and without impairing MRI visibility. We therefore need not rely on prediction to ensure safe clinical operation. Future implementations may modulate specific absorption rate (SAR) based on temperature feedback.

## Background

Interventional cardiovascular MRI has potential as a radiation-free alternative to conventional X-ray guided catheterization, but has undergone only limited clinical investigation for lack of safe and conspicuous catheter devices[1]. A guidewire is a fundamental tool for catheter-based procedures. Engineering a safe and conspicuous MRI guidewire is especially challenging because of the combination of electrical and mechanical requirements and size constraints.

A key problem is radio frequency (RF) induced heating of guidewires that contain conductive materials [2-6]. The resonant length of the conductor is a main contributor to heating, but reported values at 1.5 T are inconsistent [7,8]. Partial guidewire insertion from air into gel phantoms creates complex resonance patterns that are difficult to model. Insertion length also varies dynamically during clinical use as the operator moves the device. While detuning circuitry can mitigate inductive coupling between the guidewire and RF transmitter, capacitive coupling is more difficult to suppress. We propose a solution that allows the system or operator to recognize and respond to heating that is not suppressed by accompanying detuning circuitry.

A second problem is visibility. Commercial guidewires used in X-ray based procedures contain metal cores and radiopaque coils at the distal tip to increase fluoroscopic visibility. The interventionist needs to visualize the entire shaft and exact tip location in order to navigate vascular structures safely. Passively-visualized devices [9,10] have poor contrast that is further dependent on the device orientation relative to the main magnetic field (B_o_), and often create artifacts [11] that interfere with anatomic imaging. Actively-visualized devices incorporate receiver coils [12]. Active devices incorporating loopless antennas provide uniform shaft signal but poor tip visibility, impeding safe use. Qian and colleagues improved distal signal in a loopless guidewire by tapering distal insulation [13]. However, their approach provides indistinct tip signal. Another proposed solution combines a loopless antenna shaft with a solenoid antenna tip into a single guidewire [14] using two separate receiver channels. This approach suffers from coupling between channels and manufacturing complexity. Karmarkar and colleagues used coiled copper wire to improve tip visibility [15].

We introduce a novel active MRI guidewire design concept suitable for clinical application that balances mechanical, visualization and safety considerations. First, it incorporates a real-time temperature monitor to help ensure safe operation. Second, it provides a conspicuous tip having a unique image signature with simultaneous shaft profiling. This is achieved by combining a loopless antenna shaft with a tightly wound solenoid coil connected at the distal end. Finally, the implementation has mechanical characteristics similar to popular commercial cardiovascular guidewires.

## Methods

### Guidewire design

**Figure 1:**
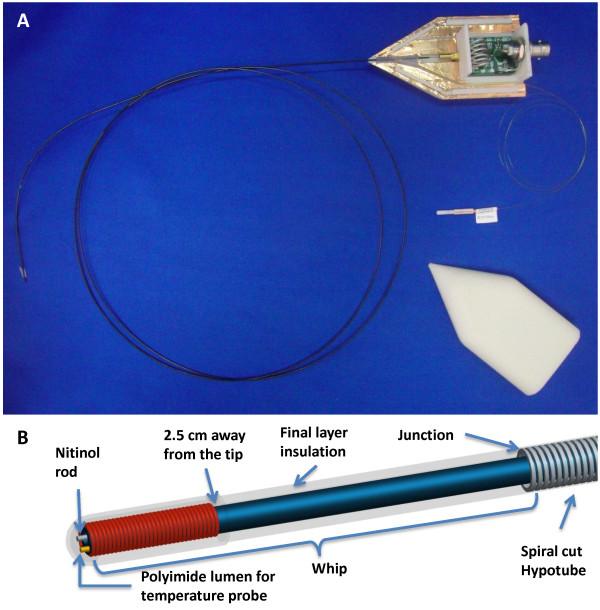
** MRI guidewire design.** A-The 0.035” guidewire has a modified loopless antenna with a tip solenoid, is 1.28 m long, and is connected to tune/match circuitry with an MMCX connector. A shielded box houses the circuitry and fiberoptic probe. B- The schematic depicts a 2.5 cm long tightly wound solenoid coil, a tapered nitinol rod, a polyimide lumen for the temperature probe, a spiral-cut nitinol hypotube for flexibility, Pebax insulation (blue) between the rod/polyimide assembly and hypotube, and a final outer layer of insulation (transparent).

The active guidewire was constructed using medical grade MRI compatible materials in an ISO class 7 cleanroom. The length was 1.28 m and diameter 0.89 mm (0.035”) for compatibility with conventional cardiovascular catheters (Figure 1A). The main shaft incorporated a modified loopless antenna with a tapered 0.25 mm diameter nitinol rod (Nitinol Devices and Components, Fremont, CA) serving as the inner conductor of the loopless antenna. Alongside this shaft was a hollow polyimide tube [0.28 mm outer diameter (OD) and 0.23 mm inner diameter (ID)] to accommodate a fiberoptic temperature probe. The distal end of the polyimide tube was closed using medical grade instant glue (Loctite, Rocky Hill, CT). Next, the nitinol rod and polyimide tube were insulated with thermoplastic elastomer polymer tubing (Pebax, Oscor Inc., FL) to ensure contact between polyimide tube and nitinol rod throughout the shaft. The sub-assembly was then inserted into a larger nitinol hypotube (0.81 mm OD , 0.66 mm ID) having a custom spiral-laser-cut pattern at the distal end to provide a smooth mechanical transition between the stiffer proximal shaft and softer distal shaft, as expected by physicians in a high performance clinical guidewire. The nitinol rod extended 12.5 cm beyond the nitinol hypotube to form a loopless antenna whip. A tightly wound copper solenoid coil (0.71 mm OD, 0.1 mm winding pitch, 25 mm long) was placed over the distal portion of the nitinol rod to manipulate current distribution on the whip. One end of the coil was electrically attached to the distal tip of the nitinol rod, and the other end was free. In effect, the whip of the loopless antenna was extended and coiled on itself. The whole guidewire assembly was insulated with a biocompatible final Pebax layer (Figure 1B). The proximal end of the guidewire connected via an MMCX type connector (Huber & Suhner, Switzerland) to matching and detuning electronics and thereafter to the MRI scanner receiver system.

The active guidewire design, including the solenoid tip, was tuned to 63.67 MHz and matched to 50 ohms using a tune/match circuit connected to the guidewire at the proximal hub. A PIN diode is activated during the RF excitation to detune the receive antenna by creating high impedance at the antenna junction where the nitinol rod extends beyond the hypotube.

### Balancing heating and tip conspicuity

Other investigators have characterized parameters affecting RF induced heating of medical devices, including length, diameter, and insulation thickness [3,16,17]. In this study, we focused on the heating implications of our enhanced-conspicuity distal tip.

First, we explored the attachment configuration of the solenoid coil to the distal end of nitinol rod. 25 mm long solenoid coils (0.71 mm OD, 0.61 mm ID) were soldered to the nitinol rods using a soldering alloy (Indium Corp., Utica, US). We considered four different solder attachment configurations of the solenoid to the rod: either the (1) distal or (2) proximal end of the solenoid attached leaving the other end unattached; (3) both ends attached; or (4) neither end attached. “Unattached” ends were suspended by insulation. We tested heating and imaging for each configuration.

Next, we explored the impact of solenoid length and diameter. The inductance of a solenoid coil is known to depend on length, cross-sectional area and the number of turns per unit area, each of which impacts signal and heating performance. We tested solenoid lengths from 10 to 50 mm and solenoid outer diameters from 0.61 to 0.81 mm to assess signal strength and RF heating.

The MR signal detected using candidate configurations was tested in a 20 × 15 × 10 cm water phantom. MRI at 1.5 T (Espree, Siemens, Erlangen, Germany) used a 3D balanced steady state free precession (bSSFP) sequence and the following typical parameters: repetition time (TR)/echo time (TE), 872.63/2.04 ms; flip angle, 45°; slice thickness, 1 mm; field of view, 340×340mm; matrix, 192×144. The ratio of the maximum tip signal to maximum shaft signal was calculated from regions of interest drawn on maximum intensity projection images.

### Comparison of internal and external fiberoptic temperature probes and in vitro heating measurements

The guidewire incorporated a dedicated polyimide port for an internal 0.15 mm diameter fiberoptic temperature probe (OpSens, Quebec, Canada). The temperature measurement system has 0.3°C system accuracy. The polyimide distal tip was sealed using acrylic glue (Loctite, Rocky Hill, CT).

We also sought to establish this internal probe always reflected equal or higher values than temperature at the guidewire surface in contact with the patient. Therefore, we temporarily affixed an external polyimide tube (0.28 mm OD and 0.23 mm ID) to the surface of the guidewire using polyester heat shrink tubing (Advanced Polymers, Salem, NH), to accommodate a second moveablex external temperature probe. The fiberoptic temperature probe first was advanced maximally into the polyimide tube. Temperature was recorded for 30 seconds before and after MRI begins, and then both internal and external fiberoptic probes were withdrawn manually to obtain a longitudinal heating profile. Locations of heating maxima were identified and compared for both inner and outer sensors. Once the hottest spot was located, the internal temperature probe was fixed to that location for further investigation.

In vitro RF induced heating tests were performed in an acrylic phantom prepared according to the ASTM 2182 standard. Guidewires were aligned parallel to the main magnetic field and fixed in position at an insertion length of 35 cm, a horizontal offset of 12.7 cm lateral to the iso-center, and a vertical offset of 6 cm from the bottom of the phantom.

The heating distribution of implants inside the ASTM phantom corresponds almost exactly to the phantom’s electrical field distribution, which increases away from iso-center [18]. Therefore, we tested the guidewire temperature at many different positions: insertion lengths 15 cm, 30 cm, 45 cm and 60 cm; depths 2 cm, 4 cm and 6 cm from the bottom; horizontal offsets 12.7 cm, 7.6 cm, 5.1 cm laterally left and right from iso-center; and at iso-center.

We used the following sequence parameters for heating experiments: bSSFP; TR/TE, 3.23/1.62 ms; flip angle, 45°; slice thickness, 6 mm; field of view, 340x340mm; matrix, 192x144; scanner reported SAR, 1.52 W/kg. For each map position, a 30 second baseline temperature recording was followed by a one minute scan, and maximum after one minute was subtracted from the baseline average.

### *In vivo* experiments

Experiments were approved by the institutional animal care and use committee and followed contemporary NIH guidelines. Two naïve Yorkshire swine (weight range 51–64 kg) underwent general anesthesia and transfemoral artery access using a 7Fr x 12 cm (14 cm including the hub) introducer sheath (Fast-Cath, St. Jude, Minnesota). Both the inner and outer temperature probes were fixed at the longitudinal hot spot identified during phantom experiments. After a 30 second baseline measurement, temperature was recorded in real time as guidewires were advanced or withdrawn manually, from the iliac artery to the aortic arch, during bSSFP MRI with TR/TE, 3.23/1.62 ms; flip angle, 45°; scanner reported SAR, 1.01 W/kg.

To increase the dynamic range of heating beyond clinical use conditions, and to better assess the relationship between inner to outer temperature measurements, the experiment was repeated with TR/TE, 3.23/1.62 ms; flip angle, 90°; scanner-reported SAR value, 3.96 W/kg. A “worst-case insertion length” was identified, and RF induced heating was measured during a one minute scan without moving the guidewire.

### Mechanical properties

The whip flexibility of the final device was compared to a commercial 0.035” nitinol guidewire (Glidewire 035, Terumo, Tokyo, Japan). To assess the flexibility of the distal 12.5 cm whip, we measured the deflection force required to achieve a range of deflection angles on a fulcrum located 1 cm, 3 cm, or 5 cm from the tip using a load cell (IDTE, Machine Solutions, Arizona).

## Results

### Balancing heating and tip conspicuity

**Figure 2:**
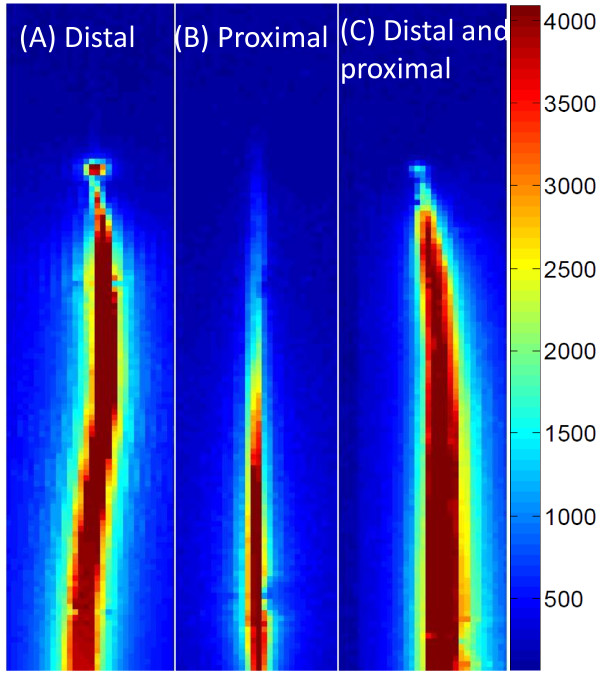
** Three different soldering configurations of the distal guidewire solenoid.** A tight-pitch solenoid coil (25 mm long, 0.71 mm outer diameter) is electrically connected **(A)** at the distal end only, **(B)** at the proximal end only, and **(C)** at both ends. The distal-only attachment creates a unique tip signal profile with a null separating it from the shaft.

Figure 2 and Table 1 show the signal profile of several candidate guidewire designs, comparing different solenoid solder configurations. A distal solenoid solder point assured a conspicuous tip signal, even if combined with a second proximal solder point. A distal-only solder point provided a maximal tip/shaft signal intensity ratio, reduced by half by soldering both the distal and proximal ends of coil, and abolished in other configurations. The distal-only design also had an image signature that best distinguished the tip from the shaft, having a signal null separating the shaft profile and the tip “point” (Figure 2A). Solder locations also impacted the position and intensity of maximum heating spot along the shaft. The highest heating was found when the solenoid was soldered proximally, less when soldered distally, still less with combined proximal and distal soldering and least when the solenoid was not soldered to the inner rod. The location of heating was spatially distributed near or between solder points. Overall, we found the best balance of distal-tip conspicuity and heating in the distal solder-point configuration.

**Table 1:** Relative signal and heating performance among guidewire designs

**Solder attachment configuration**	**Tip/ shaft signal**	**Max. heating ratio**	**Max. heated point**
**Distal**	0.97	0.63	2.5 cm away from tip
**Proximal**	0.12	1	2.5 cm away from tip
**Distal & Proximal**	0.52	0.32	1 cm away from tip
**Not Connected**	No tip signal	0.10	Junction, 12.5 cm from tip

The four rows indicate four different solder configurations of the distal solenoid: distal, proximal, both, or free-floating. For each design, the table indicates the relative signal of the tip compared with the shaft; the maximum temperature increase normalized to the worst-case design; and the location of the point of maximum heating.

**Figure 3:**
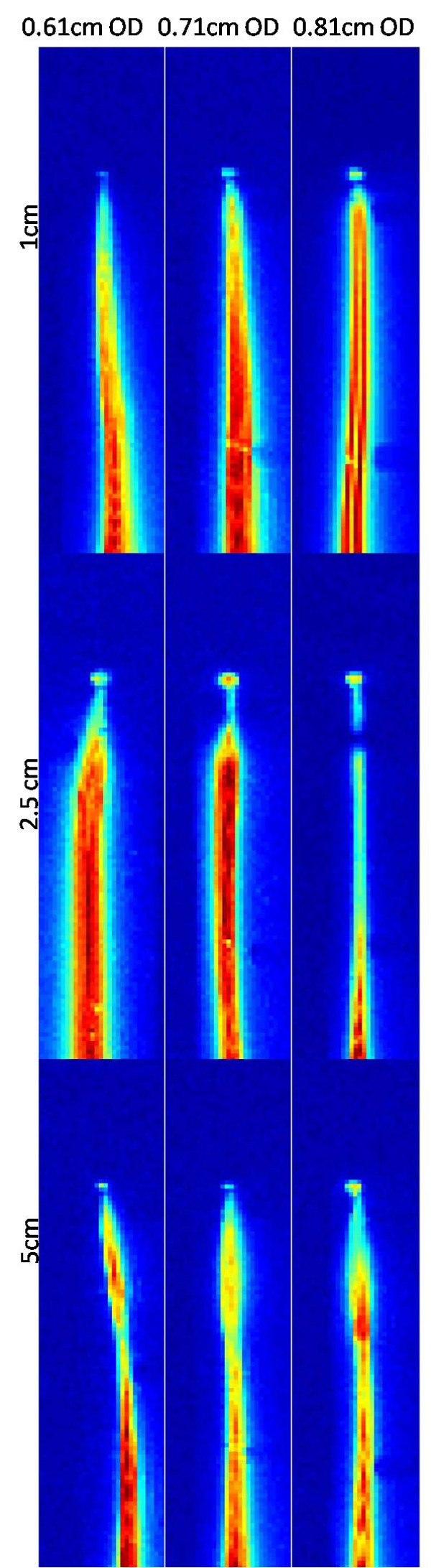
** SNR maps of guidewire (distal tip solder) for 0.61 cm, 0.71 cm, 0.81 cm coil diameter and 1 cm, 2.5 cm, 5 cm coil length configurations.** Increasing coil diameter results in brighter tip signal. Increasing coil length changes the shaft signal profile and tip signal intensity.

Next, we empirically tested different solenoid lengths (10 to 50 mm) and diameters (0.61 to 0.81 mm) to optimize the tip signal strength and heating with the distal connection. Table 2 shows tip signal normalized to shaft signal, and maximum heating location and magnitude, also depicted in Figure 3. Overall we found the most attractive solenoid configuration to be 0.71 mm in diameter and 25 mm in length with inductance 745uH for 63.67 MHz.

**Table 2:** Tip/shaft signal ratios and maximum heating

**Solenoid length**	**Solenoid diameter**		
	**0.61 mm**	**0.71 mm**	**0.81 mm**
**1 cm**			
**Tip/shaft signal ratio**	0.27	0.33	0.55
**Maximum heating (°C)**	0.27	0.26	1
**2.5 cm**			
**Tip/shaft signal ratio**	0.53	0.74	0.69
**Maximum heating (°C)**	0.55	0.59	0.25
**5 cm**			
**Tip/shaft signal ratio**	0.38	0.4	0.65
**Maximum heating (°C)**	0.15	0.16	0.22

Maximum tip to shaft signal intensity ratio and maximum heating are depicted among all soldering configurations. The heating values are normalized to the highest values achieved with the 1 cm, 0.81 mm design.

### Inner and outer temperature probe comparison

**Figure 4:**
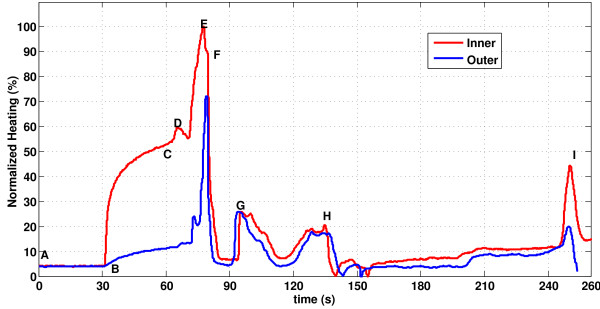
** Temperature distribution on guidewire during MR scan.A** heating profile is obtained by moving temperature sensors within (red) and on the surface of (blue)the guidewire. The probes begin beyond the guidewire distal tip. Segment A-B indicates a 30 second baseline recording; MRI begins at **B**; a relative steady-state is achieved at **C**, when probe pullback begins. Temperature peaks correspond to physical landmarks including: **D**, the distal end of the solenoid coil; **E**, the guidewire “hotspot;” **F**, the proximal end of the solenoid coil; G, the inner rod-hypotube junction point; H, gel phantom entry point; H-I, air; I, hypotube-MMCX connector solder point.

Figure 4 shows a representative temperature profile along the shaft of the guidewire, obtained by continuous probe pullback during MRI in vitro. For each guidewire prototype, we identified a hot spot within the distal solenoid.

The inner temperature was consistently higher than the outer temperature measurement, which indicates that it can adequately represent potential heating hazard. The ratio of maximum outer to inner temperature is 0.81±0.06.

**Figure 5:**
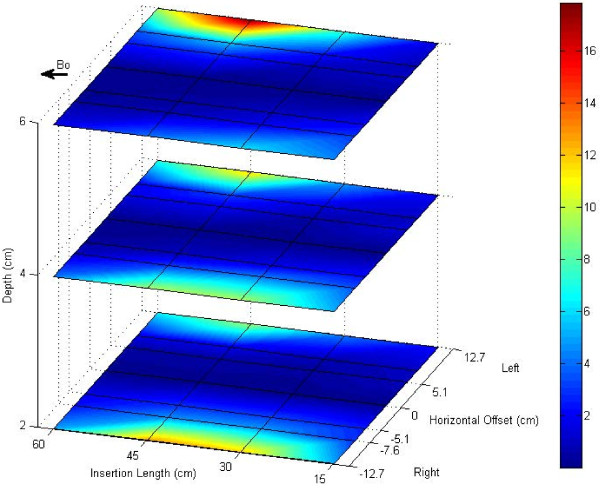
** Heating map of ASTM F2182 phantom.** The heating distribution of the guidewire in the ASTM F2182 phantom agrees with previously reported E-field distributions in the ASTM phantom. Significant heating (18°C) is observed when the guidewire is inserted into high E-fields.

The heating map of ASTM phantom was calculated by linearly interpolating sparsely acquired heating data, Figure 5. Temperature increase at the hot spot after a one minute scan was below 4°C for guidewire configurations close to the center of the phantom where the E-field magnitude is minimal. However, the temperature increase reached 18°C when the guidewire was placed at the edge of the phantom where the E-field is large.

### In vivo imaging and heating

**Figure 6:**
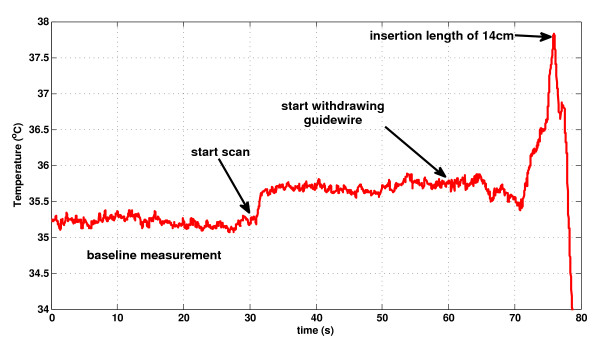
** A representative in vivo temperature recording during manual pullback.** The flip angle is increased to 90°; SAR, 3.96 W/kg to exaggerate heating. A temperature baseline is obtained before scanning begins. Scanning generates a small temperature rise. After a further interval, the guidewire is withdrawn from the aortic arch during bSSFP MRI. Noteworthy heating was observed just as the guidewire tip entered the tip of the vascular introducer sheath at an insertion length of 14 cm.

No detectable *in vivo* temperature increase was observed during MRI using a 45° flip angle; rather, a flip angle of 90^o^ was required clearly to detect a temperature increase. Figure 6 shows a representative temperature recording during manual guidewire pullback (with the temperature probe fixed at the hot spot), from the aortic arch to the iliac introducer sheath. The maximum temperature increase was observed at an insertion length of 14 cm, at which point the tip of the guidewire was just inside the tip of the sheath. In this condition, the hot spot remained 25 mm inside the sheath and was isolated from cooling blood flow. Pulling the guidewire further from 14 cm to the hub of the sheath caused a sudden temperature drop due to the temperature gradient inside the sheath, where the temperature approaches room temperature (22°C) near the hub.

**Figure 7:**
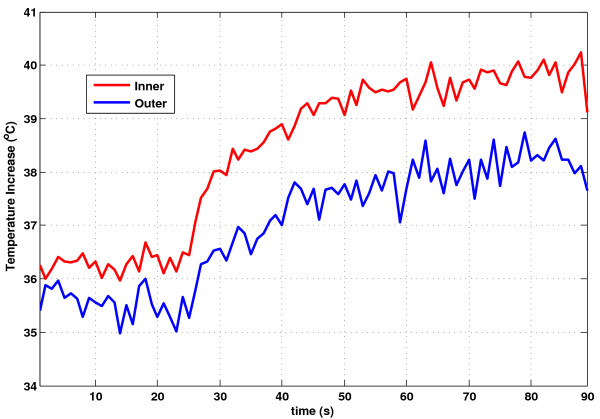
** In vivo heating measurement.** Inner (red) and outer (blue) temperature measurements of the “hot spot” at the worst-case (14 cm) insertion length, at which the guidewire remained inside the introducer sheath, using a 90° flip angle, SAR, 3.96 W/kg.

Figure 7 shows inner and outer temperature measurements on the guidewire at the worst-case insertion length of 14 cm and flip angle, 90°; SAR, 3.96 W/kg, during a one minute scan. At baseline, the average inner and outer temperatures from the two animal experiments were 36.2 ± 1.2°C and 35.7 ± 1.5°C, respectively. At the worst-case insertion length, the temperature increased by 4.2±0.3°C (inner) and 3.4±0.2°C (outer) after one minute scans at the hot spot. The ratio of outer to inner temperature was 0.80±0.03. With further advancement of the wire beyond the tip of the sheath, the temperature fell rapidly and remained within a maximum of 0.7±0.1°C over baseline, measured at the inner probe.

**Figure 8:**
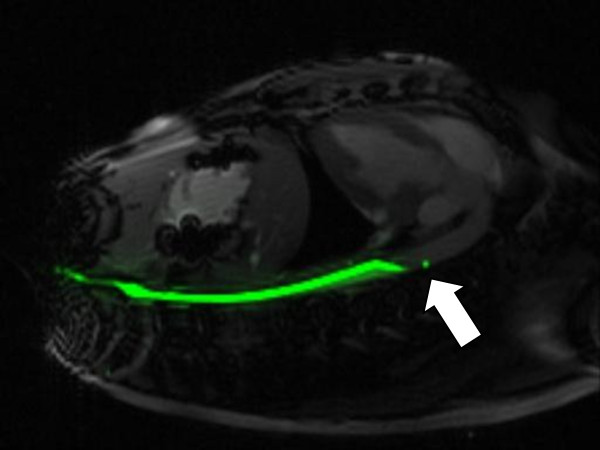
** In vivo image of guidewire in aorta of swine.** Guidewire has a conspicuous signal at the tip (white arrow).

Figure 8 shows real-time MRI of the guidewire inside a pig aorta. It is attached to a separate receiver channel and reconstructed in green. The guidewire tip appears as a distinct green point, and the guidewire shaft has a uniform signal distribution.

### Mechanical properties

**Figure 9:**
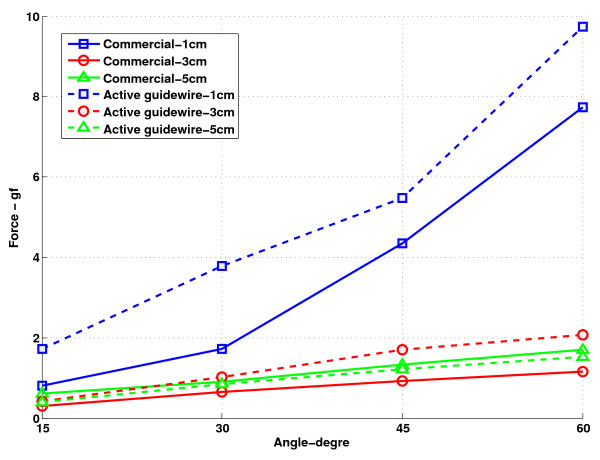
** Tip flexibility results of commercial comparator (Terumo Glidewire 035) and the active guidewire.** The active guidewire has similar flexibility characteristics to the commercial comparator. The force required to deflect the active guidewire is slightly higher than the force required to deflect the commercial guidewire.

Figure 9 shows the results of tip flexibility testing. The distal 5 cm of the active guidewire has comparable flexibility to the commercial comparator PTFE-coated nitinol guidewire (Glidewire 035, Terumo, Somerset, NJ).

## Discussion

We describe the integration of a fiberoptic temperature probe inside a compact and conspicuous guidewire that is mechanically suitable for invasive and interventional cardiovascular MRI procedures. The probe provides continuous monitoring to allow rapid detection of deleterious device heating during use, without impairing mechanical guidewire operation, and without impairing MRI visibility. Indeed the embedded fiberoptic probe, operated in pullback mode, also allowed rapid empirical testing of guidewire designs to balance heating and conspicuity. Using this real-time temperature monitoring system we developed a family of guidewire designs that have a single “heating maximum” along their length, typically near the tip, and consistently higher internal than external temperature. We consider this as a significant advance in interventional cardiovascular MRI because we no longer need to rely on models of dubious predictive value to ensure safe clinical operation. Moreover, in the future we can modulate the input energy during MRI excitation based on temperature probe feedback.

Multi-channel RF coils with separate transmission lines have been used in many MRI compatible interventional devices to visualize separate components of the instruments. In this study we eliminated separate transmission lines and electrically attached one end of a solenoid coil directly to the inner conductor of a loopless antenna base to manipulate current distribution on the whip. Electrical attachment of the coil to the inner rod provided a distinct signal at the tip. The location of the solenoid attachment point impacted both device signal profile and heating. We found a balance of the two when we soldered the distal end of the solenoid to the inner rod. In this configuration, the hot spot was 2.5 cm proximal from the tip of the guidewire. During real-time temperature monitoring, the temperature probe is fixed at this hot spot. In the future, this will allow us to create different guidewire prototypes with curved tips or J tips, without impairing mechanical properties of the guidewire.

Assessment of RF-induced heating of medical devices typically is performed in gel phantoms using multiple fiberoptic temperature probes affixed at locations expected to have significant heating. Electromagnetic simulations can help identify candidate locations, but are not readily applied to more complex mechanical designs. We therefore introduced a fiberoptic temperature probe intended to operate during actual use conditions, and designed a guidewire incorporating a lumen specifically for temperature monitoring. Recordings during probe pullback allow rapid whole-device assessment, and efficient comparison of designs. In our experiments, we waited only 30 seconds before each pullback, which approached but did not achieve thermal equilibrium, but which was adequate to identify important heating points (Figure 4, point E). Alternative temperature monitoring approaches include infrared cameras [19], which do not detect this range of heating in gel phantoms or in vivo, and heat-sensitive liquid crystal paint[16], which is water soluble and therefore unsuitable for aqueous gel phantoms that attempt to load the RF excitation coils.

While we are able to detect a temperature rise when testing the guidewire in an industry standard gel phantom, the heat evidently is dissipated during normal operation (flip angle, 45°; SAR, 1.01 W/kg) in vivo when exposedx to flowing blood during left heart catheterization in pigs. Indeed, we were able to detect a temperature rise (4.2°C) in vivo only by increasing the RF transmit power beyond expected operating conditions (flip angle 90°; SAR 3.96, W/kg), and only when the hotspot was isolated from flowing blood inside the vascular introducer sheath. In general, vascular catheterization imposes favorable geometric constraints by confining the devices to relatively central body structures where the E-field magnitude is low. However, the temperature monitor provides a desirable and incremental margin of safety that can allow the operator or system to respond to unanticipated heating caused, for example, by unusual positioning or by device malfunction.

Finally, to suit the target application of MRI heart catheterization, we were able to manufacture a device with these additional features while retaining comparable tip/whip flexibility to a popular nitinol commercial guidewire. This was possible by employing several advanced material processing and manufacturing techniques including reduced stiffness Pebax coating, a flexible solenoid coil, a grinded nitinol rod, a spiral laser-cut nitinol hypotube, and a thin fiberoptic cable.

## Conclusion

We designed an active guidewire with distinct tip signal, uniform shaft signal and embedded fiberoptic temperature probe fixed to shifted “hot-spot” location to monitor RF induced heating in real time without affecting the device functionality adversely. The embedded temperature monitoring system provides real time heating information to ensure safe operation while guidewire orientation and trajectory are dynamically changed within the vasculature.

## Competing interests

The authors declare that they have no competing interests.

## Authors’ contributions

MS developed the design, built guidewires, performed experiments, analyzed the data, and drafted the manuscript. CES developed the design, built guidewires, performed experiments, and helped to draft the manuscript. JAB built guidewires and performed experiments. DNF built guidewires and performed experiments. MH performed animal experiments. AZF helped perform experiments and supervised the study. CO participated in the conception and analysis of the experiment. RJL contributed to design and analysis, helped to write the manuscript, and supervised the study. OK developed the design, performed experiments, helped to write the manuscript and supervised the study. All authors read and approved the final manuscript.
